# Burden of disease: A scoping review of HIV/AIDS and TB in occupational noise-induced hearing loss

**DOI:** 10.4102/sajcd.v67i2.669

**Published:** 2020-03-03

**Authors:** Katijah Khoza-Shangase

**Affiliations:** 1 Department of Audiology, Faculty of Humanities, University of the Witwatersrand, Johannesburg, South Africa

**Keywords:** Africa, Conservation, Disease, HIV and AIDS, Health, Hearing, Noise, Occupational, Burden, Tuberculosis

## Abstract

**Background:**

Occupational noise-induced hearing loss (ONIHL) does not occur in isolation from other influencing factors such as health conditions and illnesses like human immunodeficiency virus and acquired immunodeficiency syndrome (HIV and AIDS), as well as tuberculosis (TB). How the burden of disease influences the occurrence and/or management of ONIHL becomes a key if the goal of hearing conservation programmes (HCPs) is to be achieved within these contexts.

**Objectives:**

The purpose of this scoping review was to conduct an investigation on how the burden of disease’s influence on ONIHL is reported in literature, with a specific focus on the most prevalent diseases in South African mines – HIV and AIDS and TB.

**Method:**

A scoping review was conducted using the Arksey and O’Malley’s framework. A search was conducted in five electronic bibliographic databases and the grey literature.

**Results:**

The search procured 10 publications, with two specific to ONIHL within the South African context. In addition to the two publications specific to TB and ONIHL, findings revealed a serious gap in the evidence around the scoping review question globally. This obvious lack of investigations into the influence of these two conditions in the South African mining context raises serious implications about the responsiveness, and proactive nature of HCPs within this population.

**Conclusion:**

Considering the burden of diseases on otology and audiology is critical as certain diseases cause hearing impairment either as a primary effect, as a secondary/opportunistic effect or as a side effect of treatment options for that disease. An employee suffering from any such disease with concomitant exposure to hazardous noise levels presents an even bigger challenge to HCPs if such is not taken into consideration in the conception, implementation and monitoring of HCPs.

## Introduction

Evidence indicates that South Africa remains one of the low- and middle-income (LAMI) countries with poor health outcomes and high mortality rates linked to the unique quadruple burden of disease (Institute for Health Metrics and Evaluation [IHME], 2018; Pillay-van Wyk et al., 2016; Rohde et al., 2008). Occupational health conditions in this context occur within this unique quadruple burden of diseases; hence, they cannot be seen and managed in a manner that is dissociated from this burden of disease. This is particularly true in occupational noise-induced hearing loss (ONIHL), which has numerous influences linked to the quadruple burden of disease (Khoza-Shangase 2019a).

Freeman (n.d.) argues that it is necessary to prioritise health conditions in South Africa because of the reality that there are limited resources, including financial and human resources, to address all health challenges. This author asserts that it is therefore crucial to focus energies and resources, otherwise nothing could be carried out properly to address healthcare challenges in the country. Putting additional resources into priorities and getting the systems and processes in place for a limited number of conditions could make a difference than trying to do everything at once, and this is the position adopted in the current scoping review where within the context of ONIHL, human immunodeficiency virus (HIV)/acquired immunodeficiency syndrome (AIDS) and tuberculosis (TB) have been prioritised as key diseases in the quadruple burden of diseases relevant within the context of South African mining industry. Adams, Ehrlich, Ismail, Quail, and Jeebhay (2018) support this position when they state that healthcare workers in the South African workforce are at risk of the rising TB because of their occupational activity and exposure to as members of communities with a high HIV and TB burden, with an increasing risk of multidrug-resistant TB.

Occupational noise-induced hearing loss is a globally prevalent condition, classified as the number one work-related disability, second only to presbycusis as a form of acquired hearing loss (Krishnamurti, 2009; Mostaghaci et al., 2013). This disability is characterised by a sensorineural partial or complete loss of hearing that develops gradually over a period of several years because of exposure to continuous or intermittent high levels of noise or hazardous levels greater than 85 dBA during an 8-h shift at workplace (Patel et al., 2010; Rappaport & Provencal, 2001; Ritzel & McCrary-Quarles, 2008). Evidence indicates that the influence of exposure to high levels of noise during their work life extends past their period of employment, as older people with presbycutic hearing loss have been found to suffer even more severe consequences if they have been exposed to high levels of noise during their working life (Mostaghaci et al., 2013; Ritzel & McCrary-Quarles, 2008). This is particularly established in developing countries (Chadambuka, Mususa, & Muteti, 2013; Miah, Rubya, & Kabir, 2014; Nandi & Dhatrak, 2008) where additional burden of disease complicates the presentation (Khoza-Shangase, 2019a).

Global estimates of the prevalence of ONIHL have indicated rapidly increasing numbers. Nelson, Nelson, Concha-Barrientos, and Fingerhut (2005) provided global estimates ranging between 7% and 21%, with Feder and colleagues (2017) recently revising these to be between 16% and 24%. This increase in prevalence of ONIHL has been reported to be greater in LAMI countries (Chadambuka et al., 2013; Feder et al., 2017; Strauss, Swanepoel, Becker, Eloff, & Hall, 2014). Specific to South African mines, the only evidence available confirms this conclusion, with Edwards, Dekker, Franz van Dyk, and Banyini (2011) reporting that approximately 73.2% of the workforce was exposed to noise levels above the legislated occupational exposure level of 85 dBA, and Chamber of Mines, as cited by Strauss et al. (2014), reporting that 3.1 out of every 1000 workers have ONIHL. Strauss and colleagues (2014) argue that over and above occupational noise exposure, within the South African context, these figures could be influenced by biological factors as well. These factors include smoking (Fabry et al., 2010), age, gender (Daniel, 2007), genetics (Lavinsky et al., 2016), ototoxic drugs and illnesses such as TB (Assuiti, Lanzoni, dos Santos, Erdmann, & Meirelles, 2013; Khoza-Shangase, 2019a). Recently, Khoza-Shangase (2019a) has found that goldminers with a history of TB treatment have worse hearing thresholds in high frequencies when compared to those without having TB history. Thus, this author highlights the importance of strategic hearing conservation programmes (HCPs), including ototoxicity monitoring in miners with a history of TB and HIV/AIDS, and the possible use of oto-protective/chemo-protective agents in the South African mining population (Khoza-Shangase, 2010a, 2010b, 2011, 2017).

Stuckler and colleagues (2011, 2013) argue that South Africa is amongst the countries with the highest incidence of HIV/AIDS and TB, with the highest prevalence of these diseases in the mining industry. This is because of the fact that TB within the South African context has been shown to grow dramatically, and has been correlated with the increased prevalence of HIV (Chaisson & Martinson, 2008). A report by Reddy and Swanepoel (2006) has stated that in the South African mining sector, approximately one-third of mineworkers acquired HIV within 18 months of being employed at the mines. Another report by AngloGold Ashanti (2012) West Wits mining district provided estimates of approximately 85% of their employees with a diagnosis of TB and HIV. Owing to the documented evidence of the compounding impact of synergistic effects of concomitant exposure to ototoxic medications used to treat HIV/AIDS and/or TB and noise (Khoza-Shangase, 2019a; Valente, Hosford-Dunn, & Roeser, 2008), the implications of the coexistence of these burden of diseases within a chronic noise exposure environment require investigation, quantification and deliberation within HCPs.

Despite the significant progress made through various programmes, such as roll out of antiretroviral therapy (ART), to reduce deaths related to HIV and improve life expectancy (Gueler et al., 2017; Joint United Nations Programme on HIV/AIDS [UNAIDS], 2018), HIV continues to increase, particularly in LAMI countries with increasing evidences of new infections (World Health Organisation [WHO], 2018). In South Africa, the prevalence of HIV almost doubled from 4.25 million in 2002 to 7.52 million in 2018 (Statistics South Africa, 2018). Hence, South Africa has become an epicentre of HIV/AIDS with a combined high rate of TB co-infection, with links having been established between treatments of these conditions and ototoxicity (Khoza-Shangase, 2019b). Owing to high numbers of patients currently on long-term treatment of TB, it is said that South Africa is ‘…potentially facing the risk of a significant proportion of the population acquiring aminoglycoside-induced permanent hearing loss’ (Bardien, Schaaf, Fagan, & Petersen, 2009). In 2017, a News24 article by Singh (2017) reported that 123 TB patients in KwaZulu-Natal (KZN) had gone deaf following TB treatment. In this article, the reason cited for the patients going deaf was negligence by the Department of Health: ‘… as their TB treatment was not properly monitored causing a complete loss of hearing’.

As part of HCPs, it is important that monitoring and management of ototoxicity in HIV/AIDS and TB is added as an important aspect of hearing conservation strategy. The need for audiometric testing to identify early changes in hearing thresholds resulting from drug therapy is felt by this population, giving careful cognisance to the concomitant exposure to excessive occupational noise. Within HCPs, if ototoxicity monitoring does not form part of the treatment, employees’ hearing outcomes could be far worse (Khoza-Shangase, 2019a) as they would not be benefitted from various treatment options, including alternative drugs, reduced dosages or altered treatment regimens, although ototoxicity is detected early during the treatment period (Lonsbury-Martin & Martin, 2001). Furthermore, removal of employee from noisy areas during ototoxic treatment to avoid the synergistic impact of noise and medications on the ear is not carried out, thus exposing the worker to higher risks.

## Methods

Adhering to the methodology advocated by Levac, Colquhoun, and O’Brien (2010), the research team comprising two researchers working in the field of preventive audiology, which includes ONIHL and HCPs, agreed on a broad research question to be addressed by scoping review and overall study protocol, including specification of search terms/keywords/phrases as well as selection of databases to be searched. The framework adopted was that of Arksey and O’Malley (2005), and it specifies five key phases: (1) identifying the research question, (2) identifying relevant publications, (3) study selection, (4) charting the data and (5) collating, summarising and reporting the results.

### Research question

The broad question that directed the current review was, ‘Does ONIHL research consider the burden of HIV/AIDS and TB as a potential influence in HCPs, and what has been documented in the literature on this?’ This question was guided by the high prevalence of HIV/AIDS and TB in South Africa and particularly in the mining sector of South Africa. The researcher aimed to perform this review to integrate and map available evidences for identification of existing lacunae that could be influencing the success or lack thereof of HCPs within the South African mining industry. Furthermore, influenced by Daudt, van Mossel, and Scott (2013) on the value of scoping reviews, the current review also revealed the types and sources of evidence available on the above-mentioned question; all of these reviews would have implications for academic and clinical training, clinical practice, policymaking as well as future research.

### Data sources and search strategy

The initial search was carried out on 18 April 2019 in the following five electronic databases: Science Direct, PubMed, Scopus Medline, ProQuest and Google Scholar. The databases were selected to be comprehensive and to cover publications considering the influence of the burden of disease (HIV/AIDS and TB) in ONIHL and HCPs. The selected studies were restricted to the studies published in English, with a focus on these two major burdens of diseases in this population. The search consisted of the following terms: Africa, conservation, disease, HIV/AIDS, health, noise-induced hearing loss, occupational, burden, impact, effects, developing countries, mines and tuberculosis.

Applying the same search process/string that was used in Science Direct, PubMed, Scopus Medline and ProQuest, a web search was conducted in Google Scholar to identify grey literature. Then *a priori* decision was made to screen only the first 10 hits (as sorted by relevance by Google Scholar) after considering the time required to screen each hit and because it was believed that further screening was unlikely to yield more relevant articles (Stevinson & Lawlor 2004). The following websites were also searched manually: The Mine Health and Safety Council; Minerals Council South Africa and Department of Mineral Resources.

The following citations, as shown in Table 1, were eventually included: Adams et al. (2018); Barwise, Lind, Bennett, and Martins (2013); Brits, Strauss, Eloff, Becker, and de Swanepoel (2012); Edwards (2009); Eisler (2003); Elgstrand and Vingård (2013); Khoza-Shangase (2019a); Kistnasamy et al. (2018); Minerals Council of South Africa (2018) and Sibanye Gold (2018). Snowball sampling was adopted where citations, meeting the inclusion criteria, within articles were searched. Another search of the above-mentioned bibliographic databases and grey literature was conducted in August 2019 to ascertain if there were any additional publications post the initial search. No new hits were identified.

**TABLE 1:** Studies reflecting burden of disease in occupational noise-induced hearing loss and its management.

Author(s) and date	Publication title	Publication focus/aims	Methodology	Context	Results
Kistnasamy et al. (2018)	Tackling injustices of occupational lung disease acquired in South African mines: Recent developments and ongoing challenges.	This study aimed to assess developments over the last 5 years in providing compensation, quantify shortfalls and explore underlying challenges.	Review	South Africa	By the end of 2017, 111 166 miners had received compensation (of which 55 864 were for permanent lung impairment, and another 52 473 for tuberculosis [TB]); however, 107 714 compensable claims remained unpaid. Many (28.4%) compensable claims are from Mozambique, Lesotho, Swaziland, Botswana and elsewhere in southern Africa, a large proportion of which have been longstanding. A myriad of diverse systemic barriers persist, especially for workers and their families outside South Africa. Calculating predicted burden of occupational lung disease compared to compensable claims paid suggests a major shortfall in filing claims in addition to the large burden of still unpaid claims.
Barwise et al. (2013)	Intensifying action to address HIV and TB in Mozambique’s cross-border mining sector.	Reports new research from 2011 to 2012 on health-related attitudes and behaviours of Mozambican mine workers and their families, and presents an estimate of the financial burden of disease related to migrant mine work for Mozambique’s public services and migrant-sending communities.	Review	Mozambique	They recommend that the Declaration be operationalised and enforced. Practical measures should include training of health workers in migrants’ right to health; user-friendly health information in Portuguese and local languages; building the advocacy capacity of mine workers’ representatives and more attention to social, cultural and economic factors that affect migrant mine workers’ health, including better access to health information and services and livelihoods for wives, widows and orphans in communities of origin.
Brits et al. (2012)	Hearing profile of goldminers with and without TB.	To compare the hearing of goldminers with and without TB to determine the effect of TB and its associated risk profile on hearing.	Audiological and medical surveillance data of 2698 South African goldminers for 2001–2009 were analysed in a retrospective cohort design. Hearing thresholds for the air conduction frequencies (0.5 kHz, 1 kHz, 2 kHz, 3 kHz, 4 kHz, 6 kHz and 8 kHz) in both ears were analysed together with biographical and occupational data. Subjects were divided into two experimental groups (single TB treatment [n = 911] and multiple TB treatment [n = 376]) and one control group (n = 1411). Comparisons between groups included (1) change from baseline to most recent audiogram, (2) most recent hearing thresholds and (3) most recent thresholds in a subset of noise exposed and unexposed groups.	South Africa	Hearing thresholds for the TB groups were significantly (*p* < 0.01) elevated compared to the control group, after correcting for time between baseline and most recent audiogram, threshold at baseline and age at test. Pair-wise comparisons demonstrated the largest threshold differences between the control and multiple TB group. Changes in mean thresholds across TB treatment groups were independent of noise exposure. Hearing thresholds over time also deteriorated significantly more (*p* < 0.01) in workers with TB (single and multiple treatment) than in workers without TB.
Khoza-Shangase (2019a)	Hearing function of goldminers with and without a history of TB treatment: a retrospective data review	The objective of this study was to compare the hearing function of goldminers with (treatment group) and without (non-treatment group) the history of TB treatment, in order to determine which group had increased risk of noise induced hearing loss. Furthermore, possible influence of age and HIV in these two groups was examined.	A retrospective record review of 102 miners’ audiological records, divided into two groups, was conducted, with data analysed both qualitatively and quantitatively.	South Africa	Findings suggest that goldminers with a history of TB treatment have worse hearing thresholds at high frequencies when compared to those without this history; with evidence of a noise-induced hearing loss notch at 6000 Hz in both groups. Pearson’s correlations showed values between 0 and 0.3 (0 and −0.3), which are indicative of a weak positive (negative) correlation between HIV and hearing loss as well as between hearing loss and age in this population.
Eisler (2003)	Health risks of goldminers: A synoptic review.	Health problems of goldminers (decreased life expectancy; increased frequency of cancer of the trachea, bronchus, lung, stomach and liver; increased frequency of pulmonary TB (PTB), silicosis, and pleural diseases; increased frequency of insect-borne diseases, such as malaria and dengue fever; noise-induced hearing loss; increased prevalence of certain bacterial and viral diseases and diseases of the blood, skin and musculoskeletal system) are briefly documented in goldminers from Australia, North America, South America and Africa.	Review	-	In general, HIV infection or excessive alcohol and tobacco consumption tended to exacerbate existing health problems. Miners who used elemental mercury to amalgamate and extract gold were heavily contaminated with mercury. Among individuals exposed occupationally, concentrations of mercury in their air, fish diet, hair, urine, blood and other tissues significantly exceeded all criteria proposed by various national and international regulatory agencies for protection of human health. However, large-scale epidemiological evidence of severe mercury-associated health problems in this cohort was not demonstrable.
Adams et al. (2018)	Occupational health challenges facing the Department of Health: Protecting employees against TB and caring for former mineworkers with occupational health diseases.	This chapter reviews two occupational populations for which the South African Department of Health has legal responsibilities, although in different ways. These are healthcare workers at risk of TB, to which the department has responsibilities as an employer, and former mineworkers with occupational lung disease, to which the department has legal responsibilities for examination and compensation under the *Occupational Diseases in Mines and Works Act* (ODMWA).	Review	South Africa	Besides infection control and prevention measures, protection of healthcare workers requires an integrated management system that incorporates commitment from top management; comprehensive, locally appropriate and practicable policies; appropriate training; continued surveillance and the provision of comprehensive occupational health services to healthcare workers. The Department of Health has major shortcomings in fulfilling its legal mandate of providing statutory medical examinations (in addition to the treatment of TB and HIV-related disease in all public healthcare facilities). The department is also failing to adjudicate and arrange for the payment of compensation to workers with occupational lung disease. Legal, financial and managerial reform of the ODMWA system is required.
Elgstrand and Vingård (2013)	Occupational safety and health in mining: Anthology on the situation in 16 mining countries.	Book	-	-	-
Edwards (2009)	85 dBA: Is it protective enough to prevent hearing loss in South African miners?	To test the hypothesis: The occupational exposure limit (OEL), which does not take into account complex exposure patterns, may not provide adequate protection for miners’ ears.	A pilot study to evaluate impact on the inner ear used otoacoustic emissions as a measure of stress to the cochlea was undertaken. Controlled exposure to noise, heat and exercise on a group of young healthy males and females was conducted using less than the prescribed OEL for noise. Pre-exposure and post-exposure otoacoustic measurements were compared to evaluate the impact of individual and combined exposures.	South Africa	Statistically significant differences were found between the pre-exposure and post-exposure otoacoustic measurements for noise as a stressor. Exposure to other health stressors did not appear to accentuate the effect on the cochlea. The results appear to indicate that further investigation of the current OELs and the methods and aspects being measured is needed.
Minerals Council South Africa (2018)	Masoyise iTB Project: Minerals Council South Africa Masoyise iTB project data report 2018.	To track progress on concerned illnesses using the Minerals Council Health Information Management System (Minerals Council-HIMS) on the Healthsource platform. To collate data on key threshold indicators from members, validate and evaluate performance against industry health and safety milestones and produce annual reports on performance.	Data used in this report were drawn from the Minerals Council (HIMS) database and the analysis was performed using the Excel spreadsheet to reflect the performance of the industry and commodities against key indicators as reflected on the 30 April 2019. Companies upload their TB and HIV data on the system on a quarterly basis and the annualised data only at year-end. The system prioritised compliance to reporting requirements to ensure that participating companies uploaded and finalised their reports prior to analysis of data.	South Africa	Report focuses on TB and HIV testing, counselling, treatment and reporting. No mention of impacts of these conditions on ONIHL.
Sibanye Gold (2018)	Occupational health and well-being: Sibanye-Stillwater Integrated Report 2018.	Report on occupational health and well-being of their employees (individualised care).	Report	South Africa	Report includes findings on ONIHL, TB, HIV – also testing, counselling, treatment and reporting. No mention of impacts of these conditions on ONIHL.

ODMWA, *Occupational Diseases in Mines and Works Act*; HIV, human immunodeficiency virus; TB, tuberculosis; PTB, pulmonary TB; HIMS, Health Information Management System; ONIHL, occupational noise-induced hearing loss; OEL, occupational exposure limit.

### Citation management

All citations were imported into the web-based bibliographic manager Endnote. Duplicate citations were removed manually. Further duplicate citations if found were removed during title and abstract relevance screening and data characterisation of full articles.

### Eligibility criteria

The researchers adopted a two-stage screening process to evaluate the applicability of publications identified in the search. The first stage involved the inclusion of publications containing keywords and phrases and those broadly describing the influence/inclusion of HIV/AIDS and/or TB (burden of disease) in ONIHL and/or HCPs to determine and characterise the existing evidence base on the impact of the burden of disease in ONIHL. The second stage involved excluding from analysis the publications that described HIV/AIDS and/or TB and hearing loss in areas other than ONIHL; however, reference lists from these publications were reviewed to identify additional relevant publications. Owing to limited resources for translation, only English publications were included.

### Title and abstract relevance screening

**FIGURE 1:**
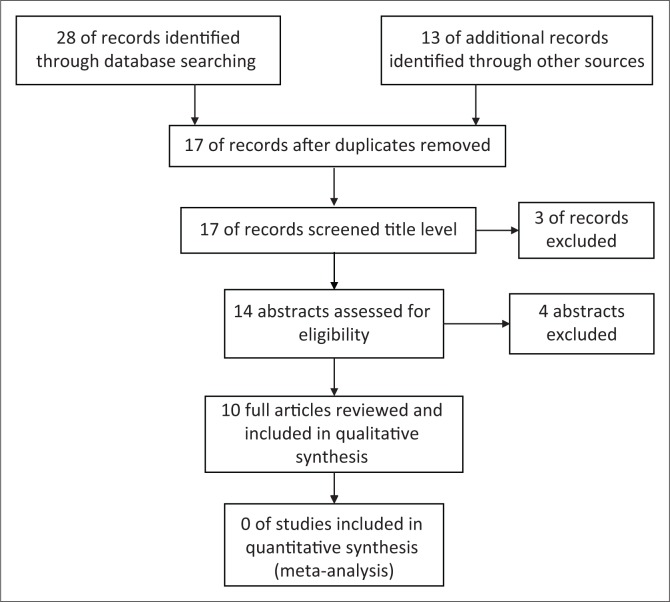
Preferred Reporting Items for Systematic Reviews and Meta-Analysis (PRISMA) flow diagram for included publications.

For efficient time management and as recommended by Arksey and O’Malley (2005), the first level of inspection of the evidences involved reviewing only the titles of the manuscripts. The second level included reviewing the abstracts only. Finally, entire articles were reviewed (refer to Figure 1). This ensured that resources were not wasted for procurement of articles that did not meet the minimum inclusion criteria of this review. The researchers used the research team’s previously developed and pretested abstract relevance screening spreadsheet, which was found to have a reviewer agreement (overall kappa) greater than 0.8 – which is considered to represent a high level of agreement (Viera & Garret, 2005). The titles, abstracts and entire articles were independently screened by both researchers. For titles where abstracts were not available, the researchers included them for subsequent review of full article during data characterisation phase. The researchers met regularly during entire process to ensure that conflicts were resolved, with author making the final decision where disagreements were found. A high level of agreement was found with the overall kappa of 0.81.

### Data characterisation

All relevant citations for the current scoping review on HIV/AIDS and/or TB in ONIHL and HCPs after title and abstract inspection were obtained for later review of full publications. A spreadsheet was developed by the author where relevance of the publication was confirmed and where details of the publication, such as type of publication, author and publication year, title, focus and aims, methodology, context, results/recommendations and reported challenges, gaps and limitations, were mentioned. The characteristics of each publication were extracted by both researchers. Further publications were excluded at this phase if they did not meet the minimum eligibility criteria. In adherence with Levac et al.’s (2010) framework, the researchers met to resolve any conflicts and ensure consistency between them as well to make sure that the publications were consistent with the set research question and purpose following their independent reviews.

### Data summary and synthesis

The data were compiled in a single spreadsheet and imported into Microsoft Excel 2016 (Microsoft Corporation, Redmond, WA, USA) for descriptive analysis.

### Ethical consideration

This article followed all ethical standards for a research without direct contact with human or animal subjects.

## Results and discussion

Current findings highlight a significant need for research in this area as indicated by paucity of evidence on highly prevalent burden of disease conditions relating to ONIHL and HCPs within the context of Africa and the world. This is despite the available evidence on the influence of HIV/AIDS and TB on hearing function within and outside the context of noise exposure (Brits et al., 2012; Cox, Reuter, Furin, & Seddon, 2017; Khoza & Ross, 2002; Khoza-Shangase, 2010a, 2019a; Matas, Angrisani, Magliaro, & Segurado, 2014; Wilson, Tucci, Merson, & O’Donoghue, 2017). The paucity was found in spite of all evidences pointing towards the synergistic relationship between noise exposure and ototoxic medications (Behar, 2018; European Agency for Safety and Health at Work [EU-OSHA], 2009; Li & Steyger, 2009). Of the publications deemed potentially relevant for this scoping review, only two South African studies were found that specifically investigated the possible influence of TB treatment on ONIHL in goldminers (Brits et al. 2012; Khoza-Shangase 2019a).

Apart from the two South African studies, nuanced analysis of publications revealed that research in occupational health has not addressed ONIHL as a complex condition that occurs in this context – a condition that does not occur in isolation, and particularly a condition that can be exacerbated by pharmacological treatments that miners receive for occupational health conditions such as TB. This conclusion is drawn from reviewed studies that address health and/or diseases in miners without linking these to each other and to occupational health prevention strategies (such as HCPs for ONIHL). These studies, as shown in Table 1, investigated the following aspects, which the current author believes have implications for the burden of disease influence on HCPs: studies on intensifying action to address HIV and TB in the mining sector; reviews on health risks amongst goldminers; investigations on challenges of occupational health facing the department of health – protecting employees against TB and caring for former mineworkers with occupational health disease; occupational safety and health in mining anthology on situations in 16 mining countries; and studies looking at whether 85 dBA as a regulation point is protective enough to prevent ONIHL in South African miners. All these publications, although speaking on the burden of disease directly and/or indirectly, are silent on focussing the influence of these on ONIHL.

Barwise et al. (2013), driven by their knowledge of the high burden of HIV/AIDS and TB in LAMI countries, conducted a study in Mozambique, where they analysed the importance of recent policy developments, both regional and national, aimed at intensifying action to address HIV and TB in Mozambique’s mining sector. These authors recommend that the country’s declaration regarding occupational health be operationalised and enforced. They further report research conducted from 2011 to 2012 on health-related attitudes and behaviours of Mozambican mine workers and their families, and present an estimate of the financial burden of the disease related to migrant mine work for Mozambique’s public services and migrant-sending communities. Furthermore, these authors suggested that practical measures, including training of health workers in migrants’ right to health; user-friendly health information in Portuguese and local languages; building the advocacy capacity of mine workers’ representatives; and giving more attention to social, cultural, and economic factors that affect migrant mine workers’ health, including better access to health information and services be put in place. These recommendations are seem to be applicable in the context of South African as well, as evidenced in Kistnasamy et al.’s (2018) study, where they explored recent developments and ongoing challenges to tackle injustices of occupational lung diseases acquired in South African mines. In their assessment of developments over the last 5 years in providing compensation, quantifying shortfalls and exploring underlying challenges with TB management in South African mines, these authors found that by the end of 2017, 111 166 miners had received compensation, of which 55 864 were for permanent lung impairment, and another 52 473 for TB. This shows significant burden on South African mining sector because of these conditions, and hence a consequent need for practical measures in ONIHL similar to those described in Barwise et al.’s (2013) study. The inferred implications of these studies for possible hearing loss-related compensation linked to noise exposure with concomitant ototoxicity are significant, with inferences for appropriate HCPs.

As far as reviews on health risks of goldminers are concerned, Eisler (2003) simply documents health problems of goldminers in Australia, North America, South America and Africa. Eisler (2003) identified noise-induced hearing loss amongst conditions such as decreased life expectancy; increased frequency of cancer of the trachea, bronchus, lung, stomach and liver; increased frequency of pulmonary tuberculosis (PTB), silicosis and pleural diseases; increased frequency of insect-borne diseases such as malaria and dengue fever; increased prevalence of certain bacterial and viral diseases; and diseases of the blood, skin and musculoskeletal system. Relationships between these health problems and their possible influence on others are not considered in this review. This is problematic as far as the question for this scoping review is concerned, and highlights the failure of mining industry to, for example, consider the affect of ototoxicity arising from treatments for most of these listed health conditions, including cancer (Landier, 2016), TB (Brits et al., 2012; Khoza-Shangase, 2019a) and bacterial and viral diseases, such as HIV/AIDS (Khoza-Shangase, 2010a, 2017, 2019b). Vulnerability of the inner ear during treatments for the mentioned diseased conditions with concomitant exposure to excessive noise has been documented to worsen presenting ONIHL (Brits et al., 2012; Khoza-Shangase, 2019a; Li & Steyger, 2009). Exploration of HCPs and health and safety regulations in the mining industry clearly indicate a gap in clinical practice and decision-making concerning management of relevant pillars of HCPs.

Of all the pillars for successful implementation of HCPs, which include periodic noise exposure measurement/monitoring; engineering controls; administrative controls; personal hearing protection; employee/management education, motivation, and training; risk-based medical examination; medical surveillance; and audiometric evaluations as well as record-keeping (Amedofu, 2007; Hong, Kerr, Poling, & Dhar, 2013), careful deliberation of employees presenting with health problems, as well as the treatment they are taking to manage these problems, is critical. Key to these ‘at risk’ employees’ HCP could be stricter administrative control, which includes reducing shifts in noisy areas; individualised or more-directed employee motivation and training programmes, including ototoxicity education and monitoring; more frequent and more comprehensive medical surveillance and audiometric evaluations. All this would require the use of a proactive data management system for record-keeping (Jantti & Cater-Steel, 2017) to be able to proactively track these ‘at risk’ miners and efficiently monitor key factors, including ototoxicity monitoring and intervention, and provide preventive and intervention measures where required.

Adams et al. (2018), in their investigations on occupational health challenges faced by the Department of Health in South Africa, recommend protection of employees against TB and caring for former mineworkers with occupational health diseases, surprisingly without any mention of the benefits of this recommendation on HCPs and miners’ hearing preservation. This publication does not mention ONIHL, and is another example of missed alignment of occupational health disease management. These authors, however, highlight Department of Health’s shortcomings in fulfilling its legal mandate of providing statutory medical examinations over and above the treatment of TB and HIV-related disease in all public healthcare facilities. The current author suggests audiological monitoring and intervention as one of these healthcare facilities. Most importantly, Adams et al. (2018) conclude their findings by sounding a crucial requirement for reform (legal, financial and managerial) of the *Occupational Diseases in Mines and Works Act* (ODMWA). On the contrary, Elgstrand and Vingård (2013), in their study on Occupational Safety and Health in Mining Anthology on situations in 16 mining countries, discussed ONIHL in isolation from the burden of diseases. This lack of viewing employees’ health conditions in a comprehensive holistic manner that acknowledges the possible influence that one condition might have on another (specifically in this case HIV/AIDS and TB’s influence on ONIHL) is also seen in specific industry annual reports of Minerals Council South Africa (2018) and Sibanye Gold (2018). These reports clearly show intensive efforts to improve the employees’ HIV/AIDS and TB status through testing, counselling, treatment and efficient reporting. None of the reports, however, deliberate on and/or mention the possible impact that these conditions have on ONIHL in their employees. Nuanced analysis of the reporting provided in these reports shows that the data management system used by Minerals Council South Africa (2018), Minerals Council Health Information Management System, is such that it shows the employees having ‘risk factors’ for ONIHL as far as these two burdens of diseases are concerned, and therefore shows the employees in need of close monitoring in their HCPs. However, this could successfully happen only if all mines are compliant with utilising this data management tool, and the current report raises non-compliance of registered companies as one of the challenges, alongside data system quality assurance and the system’s functionality limitations.

The closest study arguing for consideration of ONIHL as a complex condition that is influenced by other factors such as chemical exposure, heat and exercise (workload), is the one conducted by Edwards (2009). It questioned whether the occupational exposure limit (OEL) of 85 dBA as a regulation point is protective enough to prevent ONIHL in South African miners. This study, however, found statistically significant differences between pre-exposure and post-exposure otoacoustic measurements for noise as a stressor when using 85 dBA, but not for other health stressors. These did not appear to accentuate effect on the cochlea. Edwards (2009) concludes by recommending further investigations of 85 dBA OEL. The current author recommends inclusion of the burden of diseased conditions that are the focus of this scoping review in such future studies. This recommendation is bolstered by the only two studies found which specifically examined the possible influence of TB on ONIHL in the context of South Africa.

Brits et al. (2012), in their study on hearing profile of goldminers with and without TB in South Africa, where they compared hearing of goldminers with and without TB to determine the effect of TB and its associated risk profile on hearing, have found that hearing thresholds for TB groups were significantly (*p* < 0.01) elevated compared to the control group, and that hearing thresholds over time also deteriorated significantly (*p* < 0.01) more in workers with TB (single and multiple treatment) than in workers without TB. These findings were recently supported by Khoza-Shangase (2019a) in a retrospective data review study, which also investigated hearing function of goldminers with and without a history of TB treatment. This recent study also investigated possible influence of age and HIV in these two groups. Findings from this study suggest that goldminers with a history of TB treatment have worse hearing thresholds in high frequencies when compared with those without this history, with evidence of a noise-induced hearing loss notch at 6000 Hz in both groups. Furthermore, findings of this study have indicated a weak positive (negative) correlation between HIV and hearing loss as well as between hearing loss and age in this population. Khoza-Shangase (2019a) uses findings from this study to highlight the importance of strategic HCPs, including ototoxicity monitoring, and the possible use of oto-protective/chemo-protective agents in this population.

## Conclusions

Human immunodeficiency virus, TB and cancer are three of South Africa’s greatest burdens of disease (Olu et al., 2015). The current scope review revealed paucity of evidence and engagement with the possible influence of HIV/AIDS and TB on ONIHL and HCPs. This is a significant worry considering that mining remains an important industrial sector in many parts of the world, and one of the key sectors in South Africa where HIV/AIDS and TB are significant burdens of disease. Globally, evidence suggests that progress has been made in terms of control and management of occupational health hazards; however, significant room exists for further improvement, especially in LAMI countries, and as far as occupational diseases are concerned for managing ONIHL. Significant room exists for reducing occupational health risks as well as managing and compensating for occupational health conditions. Occupational noise-induced hearing loss risk reduction and management requires carefully conceived and efficiently implemented and monitored HCPs. These HCPs need to be contextually sensitive, responsive and relevant. Such contextualised HCPs for South Africa call for awareness and acknowledgement of the burdens of disease in the form of HIV/AIDS and TB in the presentation of ONIHL. This awareness could only increase the likelihood of success of HCPs that have shown to be less than optimal in achieving the goals of eliminating ONIHL within this context.

Occupational noise-induced hearing loss is a complex condition influenced by a number of factors, with ototoxicity being one of the key factors. Although limited research has been conducted in the synergistic effects of noise and ototoxic medications in the mining industry, sufficient evidence exists for the audiology community to increase their focus on this population and deliberate on how to ensure successful preventive measures are in place to maintain miners’ quality of life. Hearing conservation programmes naïve of the burden of disease implications fail to ensure closer monitoring of employees, individualised administrative controls for involved miners and exploration of other protective measures for employees on ototoxic treatments such as oto-protective agents; hence, implications for policy and practice reviews in HCPs are important.
